# The Japanese Wild-Derived Inbred Mouse Strain, MSM/Ms in Cancer Research

**DOI:** 10.3390/cancers13051026

**Published:** 2021-03-01

**Authors:** Kazuhiro Okumura, Megumi Saito, Eriko Isogai, Yuichi Wakabayashi

**Affiliations:** Cancer Genome Center, Division of Experimental Animal Research, Chiba Cancer Center Research Institute, 666-2 Nitonamachi Chuouku, Chiba 260-8717, Japan; kokumura@chiba-cc.jp (K.O.); megumi.s.1634@gmail.com (M.S.); eisogai@chiba-cc.jp (E.I.)

**Keywords:** wild-derived inbred mice, *Mus musculus molossinus*, MSM/Ms, carcinogenesis

## Abstract

**Simple Summary:**

Since human studies on tumor susceptibility alleles require vast numbers of DNA samples from both cancer patients and well-matched controls, their investigation and identification in humans have been complemented using mouse models. However, a number of confounding factors are associated with this type of research, including heterogeneity, weak genetic interactions, and lifestyle habits. Inbred strains relatively recently established from wild mice are often more resistant to carcinogenic stimulation and various pathogens than standard inbred mouse strains. A Japanese wild-derived inbred mouse strain, MSM/Ms has been used to map tumor resistance loci as well as other Quantitative Trait Loci (QTL) in Japan. Furthermore, genetic tools have been developed with MSM/Ms. MSM/Ms genomic sequences are currently available, which have greatly promoted the identification of tumor resistance loci as well as genes controlling quantitative variations and provide a more detailed understanding of gene function.

**Abstract:**

MSM/Ms is a unique inbred mouse strain derived from the Japanese wild mouse, *Mus musculus molossinus*, which has been approximately 1 million years genetically distant from standard inbred mouse strains mainly derived from *M. m. domesticus*. Due to its genetic divergence, MSM/Ms has been broadly used in linkage studies. A bacterial artificial chromosome (BAC) library was constructed for the MSM/Ms genome, and sequence analysis of the MSM/Ms genome showed approximately 1% of nucleotides differed from those in the commonly used inbred mouse strain, C57BL/6J. Therefore, MSM/Ms mice are thought to be useful for functional genome studies. MSM/Ms mice show unique characteristics of phenotypes, including its smaller body size, resistance to high-fat-diet-induced diabetes, high locomotive activity, and resistance to age-onset hearing loss, inflammation, and tumorigenesis, which are distinct from those of common inbred mouse strains. Furthermore, ES (Embryonic Stem) cell lines established from MSM/Ms allow the MSM/Ms genome to be genetically manipulated. Therefore, genomic and phenotypic analyses of MSM/Ms reveal novel insights into gene functions that were previously not obtained from research on common laboratory strains. Tumorigenesis-related MSM/Ms-specific genetic traits have been intensively investigated in Japan. Furthermore, radiation-induced thymic lymphomas and chemically-induced skin tumors have been extensively examined using MSM/Ms.

## 1. Introduction

Genome-wide association studies (GWAS) of human cancer have been relatively easily performed recently because next-generation sequencing has been conducted worldwide. However, this research requires a huge number of samples from both cancer patients and well-matched controls. As a number of confounding factors are associated with GWAS, such as heterogeneity, weak genetic interactions, and lifestyle habits, a vast number of cases and controls are needed to reach statistically reliable significance [1,2]. Therefore, mouse models of cancer are frequently employed to examine the genetic mechanisms conferring cancer susceptibility and resistance. They have contributed to identifying genetic loci that increase the incidence of cancer, some of which function by itself or in combinations [3,4,5,6,7,8]. 

Relatively recently, inbred mouse strains established from wild mice were found to often be more resistant to carcinogenic stimulation and various pathogens than standard inbred mouse strains. *Mus spretus*, a European wild-derived inbred mouse strain, has been shown to exhibit dominant resistance to several cancers [9,10,11]. *M. spretus* is resistant to two-stage skin carcinogenesis initiated by the application of dimethylbenz(a)anthracene (DMBA) and promoted by the application of 12-O-tetradecanoylphorbol-13-acetate (TPA). Balmain and colleagues at the University of Glasgow performed a quantitative trait locus (QTL) analysis to map tumor susceptibility/resistance loci on F1 backcross mice using *Mus spretus* and found 15 skin tumor susceptibility loci, *Skts1-15* [9,12]. A previous study reported the presence of the *Skts1* locus in an intercross of several inbred mouse strains, like the collaborative cross recently performed [13]. A number of skin tumor modifier loci besides the *Skts* series were also identified in standard inbred mouse strains and wild-derived inbred mouse strains. The *Ptch* gene was previously reported to be a genetic modifier of spontaneous squamous cell carcinoma development induce by *Karatin5* (*K5*) *Hras* transgene in backcross mice between commonly used inbred mouse strains [14], while *Psl1-4* were reported to be modifiers of DMBA/TPA induced skin tumors in backcross mice between commonly used inbred mouse strains [15,16]. Moreover, *Skts-fp 1-3* were reported to be modifiers of DMBA/TPA-induced skin tumors in backcross mice using *PWK* mice which is a wild-derived inbred mouse strain [17]. *Skts-fp 1* was detected also in a cross using *M. m. castaneous* as well [18]. 

There are currently four subspecies of *M. musculus*: *M. m. domesticus*, *M. m. bactrianus*, *M. m. musculus*, and *M. m. castaneus* [19]. An additional subspecies, *M. m. molossinus* has been identified in Japan; however, since SSLP (Simple Sequence Length Polymorphisms) analysis revealed *M. m. molossinus* was generated by hybridization between *M. m. musculus* and *M. m. castaneus*, it is not a novel subspecies [20]. Shiroishi and colleagues at the National Institute of Genetics (Shizuoka, Japan) previously determined the sequences of the two genomes of *M. m. molossinus*–derived inbred strains, MSM/Ms (MISHIMA/Mishima) and JF1/Ms (Japanese Fancy Mouse 1/Mishima). MSM/Ms was established from Japanese wild mice and the ancestry of JF1/Ms was originally found at the street market in Europe and then brought to Japan because the mouse had black spots on the back and looked similar to the mice which appeared on an old book published in the Edo era [21]. These sequences were compared with the C57BL/6J (C57BLACK/6Jackson) reference sequences and the sequences of 17 inbred strains. The results clearly showed that genome incorporation occurred from *M. m. molossinus* into *M. m. domesticus*, which contributed to produce the basic genomic structure of classical inbred mouse strains. Moreover, long segments were found to be shared in the genomes between classical inbred mouse strains including C57BL/6J and JF1/Ms mice.

The MSM/Ms strain was established from *M. m. molossinus*. The first group of mice was captured in one citizen’s house in Mishima, Japan. After several rounds of mating, those mice were donated to Dr. Moriwaki at the National Institute of Genetics (Mishima, Japan) in 1978 [22]. They started sister-brother mating in the institute. Inbreeding has reached generation N100 and, thus, these mice are now recognized as an inbred mouse strain like other commonly inbred mouse strains. Due to its genetic divergence from standard inbred mouse strains mainly established from *M. m. domesticus*, MSM/Ms has been broadly used in linkage studies mainly in Japan. A bacterial artificial chromosome (BAC) library has been constructed for the MSM/Ms genome, and sequence analysis of the MSM/Ms genome showed approximately 1% of nucleotide sequences differed from those in the standard inbred mouse strain, C57BL/6J [23,24]. Therefore, the MSM/Ms strain has been used for investigations of gene functions including SNPs (Single Nucleotide Polymorphisms). These genomic sequence differences make phenotypes of MSM/Ms mice very different from those of commonly used inbred mouse strains; they have a smaller body size [22], resistance to high-fat-diet-induced diabetes [25], high locomotive activity [26], and resistance to age-onset hearing loss [27,28], inflammation [29], and tumorigenesis [22,29,30,31,32,33,34,35,36] (Table 1). Furthermore, ES cell lines established from MSM/Ms enable us to manipulate the MSM/Ms genome [37]. Therefore, genomic and phenotypic analyses of MSM/Ms reveal novel insights into gene function that have not been possible using common laboratory strains.

We herein focused on the resistance of MSM/Ms to cancer. As we mentioned earlier, inbred mouse strains recently derived from wild mice are often resistant to carcinogenic stimulation. Mus musculus inbred strains have significantly longer telomeres than mouse strains derived from wild mice [38,39]. It is still necessary to map the stable inheritance of telomeres and to assess the expression of telomerase in the MSM/Ms strain. Since it is a hybrid between *M. m. musculus* and *M. m. castaneus*, it may have implications for cancer modeling. Radiation-induced thymic lymphomas and chemically-induced skin tumors have been extensively examined using MSM/Ms. We will mainly review these two types of tumor studies using MSM/Ms.

## 2. Radiation-Induced Thymic Lymphomas

### 2.1. Identification of Tumor Resistance Loci

**Figure 1:**
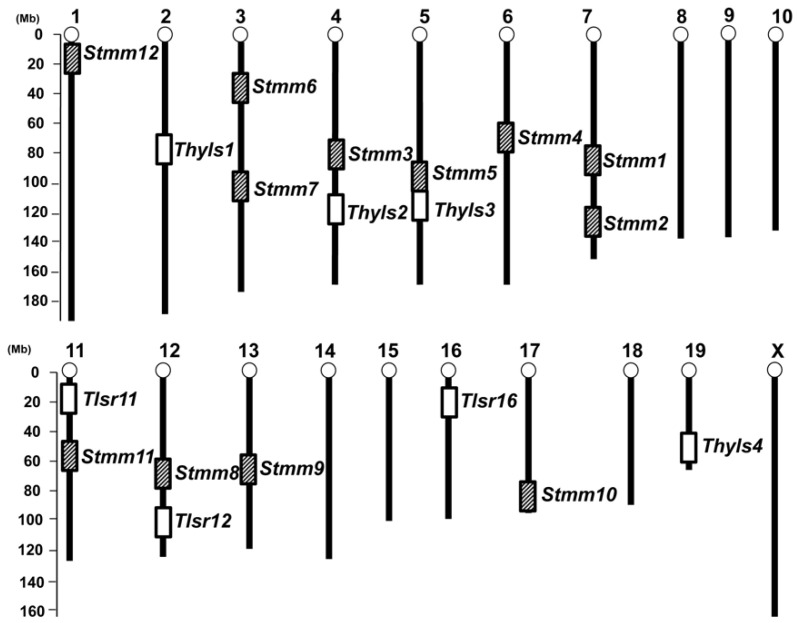
Tumor resistance (susceptibility) and LOH (Loss of Heterozygosity) regions identified in skin and radiation-induced tumors with MSM. *Stmm*; Skin tumor modifier of MSM, *Thyls*; Thymic lymphoma susceptibility, *Tlsr*; Thymic lymphoma suppressor region. Shaded squares indicate *Stmm* loci identified in skin. White squares indicate *Thyls* and *Tlsr* loci identified in thymic lymphomas. Black bars indicate each chromosome.

Resistance to radiation-induced thymic lymphomas is dependent on mouse strains [40,41] (Table 2). BALB/c (Bagg ALBINO/c genotype at the color locus) is a susceptible strain to the radiation-induced mouse thymic lymphomagenesis, whereas MSM/Ms is resistant. The incidence of radiation-induced thymic lymphomas in F1 mice is similar to that in BALB/c, indicating that BALB/c exhibits dominant susceptibility to these lymphomas. Kominami and colleagues at Niigata University (Niigata, Japan) examined an association with the control of lymphoma susceptibility of backcross mice between these two strains to identify the genetic loci involved [33]. Three markers, D2Mit15 (*Thyls1* for Thymic lymphoma susceptibility 1), D4Mit12 (*Thyls2*), and D5Mit5 (*Thyls3*), showed a strong linkage (Figure 1). Mice having lymphoma exhibited more heterozygous genotypes at D2Mit15 and D4Mit12 than MSM/Ms homozygosity, indicating that BALB/c has a dominant susceptible allele in these loci, whereas MSM/Ms has resistant alleles. However, the genotype of mice with lymphoma exhibited more MSM/Ms homozygosity than heterozygosity at D5Mit5, indicating MSM/Ms has susceptible alleles in this locus. Cooperative effects on lymphomagenesis were also observed among the three loci.

In an attempt to cut down the candidate interval around D4Mit12, congenic mice carrying a 40-Mb chromosomal region on chromosome 4 from the MSM/Ms genome were generated. These mice were crossed with BALB/c mice to further divide the congenic region. As a result, four subcongenic lines were generated by genotyping with microsatellite markers [42]. Congenic mapping and a haplotype analysis suggested metal-responsive transcription factor-1 (*Mtf-1*) was a responsible gene for *Thyls2* on chromosome 4. Sequence analysis showed lymphoma susceptible strains had a polymorphism in *Mtf-1*, in which serine exists at position 424 in the proline-rich domain. In contrast, lymphoma-resistant strains were found to have proline in that position. Comparisons of the transcriptional activity of *Mtf-1* having both alleles were performed by expressing both constructs in *Mtf-1*-deficient cells, respectively. As a result, proline at position 424 conferred stronger metal responsiveness. The radiation inducibility of target genes was also stronger in resistant congenic lines having the *Mtf-1* allele of the proline type. As the target products induced by irradiation inhibited the cellular stresses, resistant congenic strains to thymic lymphomas having the proline type seemed resistant to radiation effects, which probably resulted in resistance to the development of lymphoma.

Kominami and colleagues generated congenic mouse lines covering a 28.4 cM interval of *Thyls3* on chromosome 5 by eliminating genomic DNA of susceptible MSM/Ms alleles from F1 mice between BALB/c and MSM/Ms mice to confirm the susceptibility genes in the *Thyls3* region of MSM/Ms. MSM/Ms itself is a strain resistant to thymic lymphomagenesis. However, the *Thyls3* locus on chromosome 5 of MSM/Ms exceptionally conferred susceptibility to lymphomas [43]. They induced lymphomas in these congenic mice in two ways. Half of the congenic mice were subjected to irradiation, and the other half of the mice were administered *N*-methyl-*N*-nitrosourea (MNU), an alkylating agent. As a result, 87.5% of the irradiated congenic mice that are MSM/Ms homozygous at D5Mit5 developed radiogenic lymphomas. On the other hand, 46% of the irradiated congenic mice that are heterozygous at D5Mit5 developed radiogenic lymphomas. MSM/Ms homozygosity at D5Mit5 gave rise to a significantly higher frequency of radiogenic lymphomas. These results strongly suggested MSM/Ms had a strong susceptible allele in *Thyls3* on chromosome 5. In contrast, the difference in the frequencies of MNU-induced thymic lymphomas was not detected between MSM/Ms homozygosity and heterozygosity at D5Mit5. These findings indicated a susceptible MSM/Ms allele for thymic lymphomas and *Thyls3* conferred susceptibility specifically to radiation carcinogenesis, but not to MNU.

In order to identify *p53* (*Trp53*)-dependent tumor resistance genes, Kominami and colleagues also attempted to map genetic modifiers of radiation-induced thymic lymphomas and skin tumors in *p53* knockout MSM/Ms mice [44]. *p53^+/−^* backcross mice were generated by crossing *p53* knockout MSM/Ms mice with FVB/N mice and were subjected to radiation to induce a large number of lymphomas and skin tumors. Genome-wide screening exhibited BALB/c alleles at D19Mit5, D19Mit90, and D19Mit123 on chromosome 19 prolonged the latency of the development of thymic lymphoma and the survival of the mice (*Thyls4,* Figure 1). These results indicated that BALB/c alleles in the *Thyls4* region conferred resistance to radiation-induced thymic lymphomas and MSM alleles in that region conferred susceptibility on *p53^+/−^* background. This is the same situation as shown in *Thyls3* on chromosome 5, as mentioned earlier. D19Mit90 and D19Mit123 also showed the linkage to the latency of skin tumors and survival, whereas D19Mit5 did not show the linkage. In order to confirm the linkage at the three markers to the development of thymic lymphomas, they crossed *p53^+/−^* BALB/c mice with consomic mice having chromosome 19 of MSM/Ms on the BALB/c background. These mice were subjected to radiation and the linkage study was carried out. As a result, D19Mit90 exhibited a significant linkage as well as in the backcross study. In contrast, D19Mit5 and D19Mit123 did not show any linkage. These results suggest two possibilities. Consomic mice were originally generated on the C57BL6/J background and backcrossed to BALB/c mice several times to replace the background strain. The C57BL6/J genome could remain and mask the effect of MSM/Ms alleles of D19Mit5 and D19Mit123. The second is that the linkage at D19Mit5 and D19Mit123 were dependent on epistatic interactions. In other words, the D19Mit90 region could function by itself, whereas the D19Mit5 and D19Mit123 regions could require other regions from MSM/Ms to work in combinations.

### 2.2. Genetic Analysis of the LOH Region

Kominami and colleagues performed a genome-wide LOH analysis of radiation-induced thymic lymphomas obtained from F1 mice between BALB/c and MSM/Ms mice and backcross mice that are heterozygous for several chromosomes. Approximately 50% of those mice were a *p53*^+/−^ [45]. The findings obtained revealed that two loci exhibited frequent LOH and both alleles of the two loci were equally lost; one was mapped within a 2.9 cM region (*Tlsr12* for Thymic lymphoma suppressor 12) between D12Mit53 and D12Mit279, and the other was mapped near D16Mit122/D16Mit162 (*Tlsr16*) (Figure 1). D12Mit279 showed 62% of LOH frequency, regardless of the presence of the *p53*^+/−^ allele. In contrast, D16Mit122 showed 62% of LOH frequency on the *p53*^+/−^ background and 13% on the *p53*^+/+^ background, suggesting the presence of *p53*^+/−^ allele increased the frequency of LOH at D16Mit122. These results strongly suggested at least two types of tumor suppressor genes were located around D12Mit279 and D16Mit122, one was independent of *p53* and the other was dependent on *p53*.

They generated a physical map encompassing the *Tlsr12* locus with YAC and BAC clones to isolate new probes and to further narrow down the candidate interval having suspected tumor suppressor genes [46]. Allelic loss mapping with polymorphic markers from YAC and BAC clones finally revealed both alleles of two end markers of one BAC clone were retained, and one marker between the two end markers showed LOH in genomic DNA of lymphoma tissues, strongly suggesting the minimal interval of LOH on chromosome 12 was covered by the BAC clone. 

Sequence analysis of the peak LOH region was then conducted. The findings obtained showed the isolation of the *Bcl11b* gene from this region [47]. Biallelic changes were more frequently detected in *p53*-proficient lymphomas than in *p53*-deficient lymphomas, indicating inactivation of the *Bcl11b* gene is dependent on the presence of functional *p53* in the development of lymphoma. The introduction of *Bcl11b* into cultured tumor cell lines lacking the expression of *Bcl11b* exhibited a suppressive effect of *Bcl11b* on tumor cell growth. Taken together, these findings suggested that biallelic mutations in *Bcl11b* contributed to mouse lymphomagenesis on a *p53* wild-type background. 

*Bcl11b*-deficient mice were then generated by a conventional gene targeting method, in which homologous recombinant ES cells were selected in the presence of neomycin. *Bcl11b*-deficient mice exhibited neonatal death. A significant block in thymocyte differentiation at the CD4^-^CD8^-^ the double-negative stage was observed in the neonatal thymus of these knockout mice. In contrast, any impairment was not observed in cells of the B or γδ T cell lineage in thymocyte development [48]. In addition, the unsuccessful recombination of V(β) to D(β) was seen in *Bcl11b*^−/−^ thymocytes, which also lacked the pre-T cell receptor (TCR) complex due to the lack of *Tcrb* mRNA. Furthermore, markedly increased apoptosis was observed in the neonatal thymus of *Bcl11b*^-/-^ mice. These findings suggested that *Bcl11b* is essential for both differentiation and survival in the development of thymocytes. 

*Bcl11b*-deficient mice were mated with *p53*-deficient mice and subjected to radiation. *Bcl11b*^+/−^*p53*^+/−^ mice subsequently developed significantly more lymphomas than *Bcl11b*^+/+^*p53*^+/−^ mice; however, the wild-type *Bcl11b* allele was retained and expressed in the majority of lymphomas [49]. These findings suggested that *Bcl11b* was haploinsufficient for the suppression of thymic lymphomagenesis in *p53*^+/−^ mice, namely a condition under which the functional loss of only one allele was enough to confer an advantage for tumorigenesis. Furthermore, haploinsufficiency was supported by the findings that impairment in thymocyte development and survival was seen in *Bcl11b*^+/-^ mouse embryos as well as in *Bcl11b*^−/−^ mice.

Their genome-wide analysis of allelic loss for radiation-induced thymic lymphomas revealed the LOH region (*Tlsr11*, Figure 1) on the top of chromosome 11 in addition to chromosomes 12 and 16, as mentioned earlier. As mouse genome information was accumulated at that moment, the *Ikaros* gene was identified in this region from the database [50]. Fine allelic loss mapping around the *Ikaros* gene in genomic DNA of lymphomas suggested that the critical region of allelic loss was found in the middle of the *Ikaros* gene. The N-terminal zinc finger and the activation domains of *Ikaros* exhibited homozygous deletions and mutations. These findings strongly indicated *Ikaros* plays a key role in mouse thymic lymphomagenesis. Since the *Ikaros* gene showed biallelic changes at a high frequency in radiation-induced mouse thymic lymphomas, they investigated two other members of the *Ikaros* gene family, *Helios* on chromosome 1 and *Aiolos* on chromosome 11, [51]. Genetic analysis with adjacent MIT markers to the two genes suggested neither locus showed LOH in genomic DNA of the thymic lymphomas on the *p53* wild-type background. In contrast, both *Helios* and *Aiolos* loci showed LOH on the *p53* heterozygous background. These results suggested tumor-suppressive functions of *Helios* and *Aiolos* were dependent on *p53* loss.

## 3. Chemically-Induced Skin Tumors

### 3.1. Identification of Tumor Resistance Loci

Resistance to chemically-induced skin tumors is highly dependent on mouse strains [52] (Table 2). We previously reported that MSM/Ms is a dominant resistant strain to chemically-induced skin tumors when they are crossed with a highly susceptible strain, FVB/N (Friend Virus B/NIH), and treated with DMBA/TPA according to the standard two-stage skin carcinogenesis protocol [36]. In order to carry out genome-wide screening of genetic modifiers for DMBA/TPA-induced skin tumors, we generated *p53*^+/−^ or *p53*^+/+^ backcross mice between FVB/N and MSM/Ms mice. Approximately 50% of these backcross mice were *p53*^+/−^, which allowed us to screen *p53*-dependent skin tumor modifier loci as well as *p53*-non-dependent modifier loci. Genome-wide genetic modifier screening showed a significant linkage to the number of papillomas on chromosomes 6 (*Stmm4* for Skin tumor modifier of MSM/Ms) and 7 (*Stmm1, 2*) and a possible linkage on chromosomes 1 (*Stmm12*), 3 (*Stmm6, 7*), 5 (*Stmm5*), 11 (*Stmm11*), 12 (*Stmm8*), 13 (*Stmm9*), and 17 (*Stmm10*) (Figure 1). We then classified tumors into three size categories (<2 (hereafter “micro”), 2–6 (hereafter “middle”), and >6 mm (hereafter “large”)) and performed linkage analysis to identify stage-dependent linkage loci. The *Skts1* locus on chromosome 7 showed a strong linkage in mice that developed micro or middle-sized papillomas. However, we did not see any linkage on chromosome 7 in mice that developed large papillomas, whereas a linkage to large papillomas and carcinomas was seen at a different locus on the chromosome (*Stmm3*, Figure 1). *Stmm3* locus, which was detected around the *Cdkn2a/*p19^Arf^ gene showed *p53*-dependency because this locus was detected only in *p53*^+/+^ backcross mice, not detected in *p53*^+/−^ backcross mice. Furthermore, a suggestive linkage conferring “susceptibility” to carcinoma was also found on chromosome 5 (*Stmm5*). This is the only “susceptibility” locus MSM/Ms conferred in our study, although MSM/Ms itself is very resistant. These findings strongly suggested that multiple loci regulate each stage of tumorigenesis, some of them showed *p53* dependency.

### 3.2. Tumor Resistance Loci on Chromosome 7

To confirm the presence of tumor resistance loci on chromosome 7, we selected resistant backcross mice and eliminated MSM/Ms genomic DNA by backcrossing the mice to FVB/N mice in order to generate congenic mouse lines spanning the linkage region on chromosome 7 [53]. We first generated congenic mouse lines covering the *Stmm1* and *Stmm2* regions, respectively. Successive rounds of crossing and repeated DMBA/TPA chemical carcinogenesis experiments on each line allowed us to eliminate the whole *Stmm2* region from the candidate interval and to cut down the *Stmm1* region to approximately 3 cM regions. 

We then checked allelic imbalances on chromosome 7 to investigate the specific location of somatic changes in the *Stmm1* region on chromosome 7. Two allelic imbalance peaks were observed within the 3 cM region identified with the multiple congenic lines covering the *Stmm1* region. The combination of these two peak regions reduced the total physical size of the *Stmm1* region to approximately 5.4 Mb [53].

**Figure 2:**
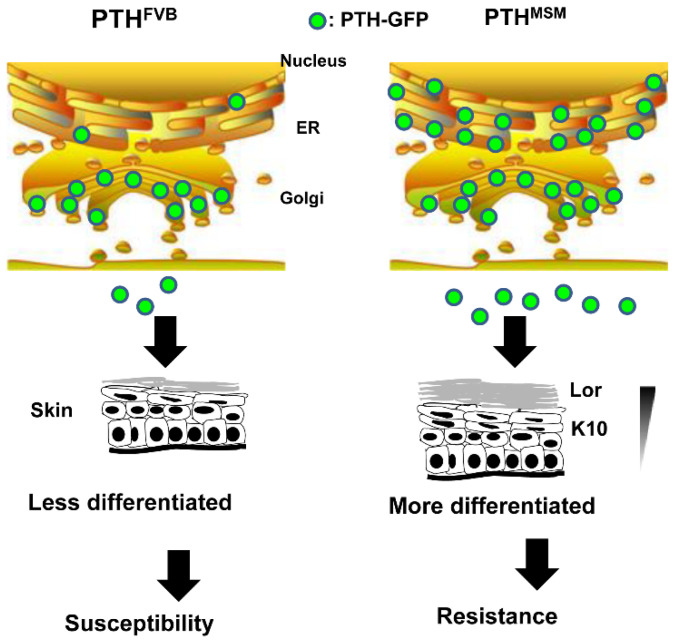
Mechanism of action of PTH in skin tumor resistance. The coding SNP in the Pro-PTH encoding region markedly enhanced the processing and increased secretion of PTH in MSM/Ms. Higher PTH levels elevated calcium levels in keratinocytes, which promoted their differentiation and ultimately led to resistance to skin tumorigenesis. ER denotes Endoplasmic Reticulum. K10 denotes Keratin 10. Lor denotes Loricrin.

We continued congenic mapping and DMBA/TPA chemical carcinogenesis and narrowed down the candidate interval of *Stmm1* to a region of 3.4 Mb on chromosome 7. *Pth* (*parathyroid hormone*) was detected among the genes mapped within the *Stmm1* region [54]. PTH is well-known to function cooperatively with vitamin D to regulate calcium and phosphate homeostasis in the blood. However, the role of PTH in skin tumorigenesis has not yet been elucidated in detail. Previous studies reported that PTH slowed down the proliferation and differentiation of cells in the epidermis, suggesting its role in skin tumorigenesis [55]. We first measured the concentration of intact PTH (iPTH) in sera of cancer-resistant MSM/MS and susceptible FVB mice. As a result, significantly higher iPTH level was detected in sera from MSM/Ms than from FVB/NJ mice. Therefore, skin carcinogenesis experiments were carried out with MSM-BAC transgenic (*Pth*^MSM^-*Tg*) and *Pth* knockout heterozygous mice (*Pth*^+/−^). *Pth*^MSM^-*Tg* mice developed a significantly lower number of tumors compared to the wild-type mice. In contrast, *Pth*^+/−^ mice developed a significantly higher number of tumors. These results strongly suggested iPTH in sera regulated skin tumorigenesis in a dose-dependent manner. Moreover, differentiation markers, such as Loricrine and Keratine 10 were highly expressed in the epidermis of *Pth*^MSM^-*Tg*, suggesting PTH promoted differentiation of cells in the epidermis. Furthermore, the in vitro experiments showed the coding SNP (rs51104087, Val28Met) in the mouse Pro-PTH encoding region enhanced its processing and secretion of PTH as well as intracellular calcium levels. Collectively, these findings demonstrated that PTH promotes terminal differentiation in keratinocytes by increasing intracellular calcium levels in keratinocytes, which results in resistance to tumors (Figure 2). 

**Figure 3:**
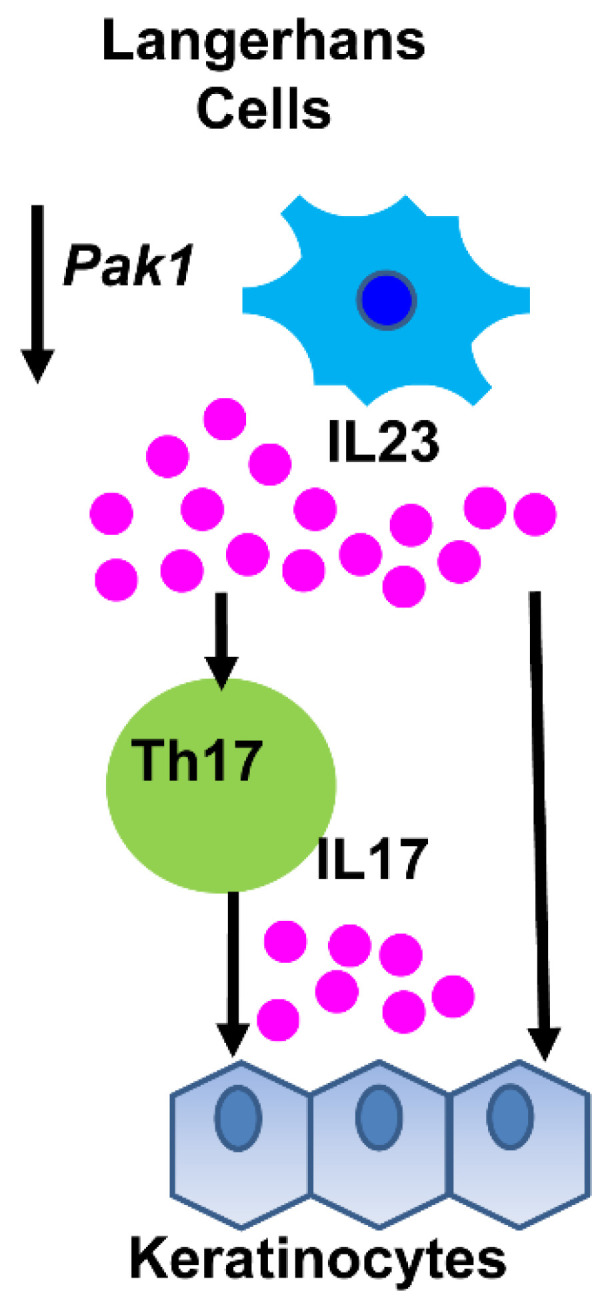
Mechanism of action of PAK in tumor resistance. PAK1 mainly localizes to Langerhans cells (LCs). IL-23 and Th 17 cell numbers were elevated in the skin of F_1_-*Pak1*^+/−^ mice. F_1_-*Pak1*^+/−^ mice had a lower number of epidermal stem cells in the skin bulge, which may be due to the abnormal proliferation of keratinocytes, resulting in tumor resistance.

*Stmm1* region on chromosome 7 was finally subdivided into *Stmm1a (about 0.24 Mb)* and *Stmm1b* (about 4.7 Mb). Both congenic lines including *Stmm1a* and *Stmm1b* exhibited a strong suppressive effect on papilloma development, respectively. *Pth* was identified in the *Stmm1b* region. *Pak1* (serine/threonine p21-activated kinases 1) was identified in *Stmm1a* region [56]. It is well-known to exhibit oncogenic activity in several cancers. Therefore, *Pak1* knockout mice were generated using ES cells from MSM/Ms with the CRISPR/Cas9 system, and DMBA/TPA skin carcinogenesis experiments were performed using *Pak1*^+/−^ F_1_ (FVB/N × MSM/Ms) mice. As a result, *Pak1*^+/−^ mice developed almost no tumor. Immunohistochemistry revealed that PAK1 was strongly expressed in Langerhans cells (LCs) as well as in keratinocytes. Furthermore, *Pak1* homozygous knockout mice on MSM/Ms background (*Pak1*^−/−^MSM/Ms^−^) showed a significant decrease in the number of LCs. F_1_-*Pak1*^+/−^ mice exhibited a decrease in the number of epidermal stem cells in the skin bulge and an increase in the number of Th17 cells in the skin. We generated *Pak1* knockdown cells using LC-derived XS52 cells (XS52-*Pak1*KD) and carried out co-culture experiments with keratinocyte-derived C5N cells. As a result, the proliferation of C5N cells was significantly enhanced in the presence of supernatants of XS52-*Pak1*KD cells. Taken together, these findings indicated that *Pak1* was required for the maintenance of epidermal stem cells, which showed an abnormal growth in the absence of *Pak1* in LCs and were not maintained correctly, resulting in the resistance to tumors (Figure 3).

### 3.3. Tumor Resistance Loci on Chromosome 4

In our previous genome-wide linkage study to map genetic modifiers conferring resistance to DMBA/TPA-induced skin tumors, *Stmm3* was found to possess loci that conferred strong resistance to larger papillomas on chromosome 4. To confirm the presence of the tumor resistance loci identified in this region on chromosome 4, we generated congenic mice harboring this region of the MSM genome on the FVB/N background [57]. DMBA-TPA carcinogenesis experiments on each line cut down physical interval to less than 34 Mb on proximal chromosome 4. We also examined somatic changes in the tumors of congenic mice to further cut down the interval. As a result, allelic imbalances, high frequencies of MSM allele loss, or FVB allele gain were detected, suggesting that a physical distance was cut down to approximately 25 Mb.

**Figure 4:**
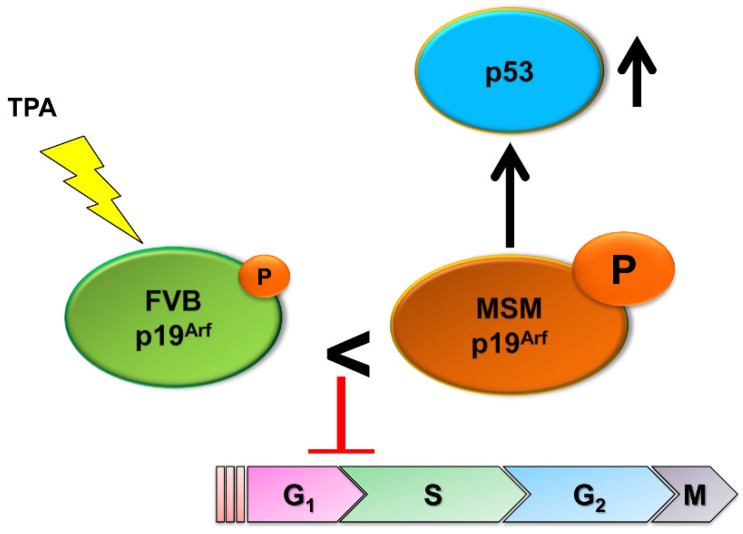
Mechanism of action of MSMp19^Arf^ in tumor resistance. The protein level of MSMp19^Arf^ is higher than that of FVBp19^Arf^ in the presence of TPA. The greater amount of p19^Arf^ more efficiently up-regulates p53 and downstream tumor suppressor genes, resulting in tumor resistance.

A congenic line with the minimal interval was crossed with *p53*^+/−^ FVB mice and these mice were treated with DMBA/TPA. The findings obtained revealed strong inhibitory effects on the development of papilloma in *p53*^+/+^ congenic mice. In contrast, papilloma development was not markedly suppressed in *p53*^+/−^ congenic mice. Therefore, the candidate gene was expected to be functionally dependent on *p53*, which was consistent with our previous findings by the initial linkage analysis of backcross mice [36]. *Cdkn2a*, a cyclin-dependent kinase inhibitor gene, was detected in the minimal interval. Two different proteins, p16^Ink4a^ and p19^Arf^ are encoded by this locus. These two proteins share exons and are produced from alternative splicing. These two well-known tumor suppressors, *p16^Ink4a^* and *p19^Arf^* were previously shown to promote the growth-inhibitory effect of pRb and p53 protein, respectively. In addition, the stimulation of separate promoters in the upstream of exon1α (encoding *p16^Ink4a^*) and exon1β (encoding *p19^Arf^*) initiated *RB*- and *p53*-dependent programs, respectively [58,59]. As our initial linkage and the congenic study suggested the gene responsible *Stmm3* was functionally dependent on *p53*, *p19^Arf^* seemed more likely to be responsible for *Stmm3* than *p16^Ink4a^*. To prove *p19^Arf^* is responsible for *Stmm3* and exclude the possibility that *p19^Arf^* is responsible, we eliminated MSM/Ms alleles of *p16^Ink4a^* and *p19^Arf^* respectively in MSM/Ms ES cell lines with CRISPR/Cas9 system and generated *p16^Ink4a^* and *p19^Arf^* knockout mice on the MSM/Ms background. These mice were crossed with FVB/N mice to generate *p16^Ink4aFVB^*^/-^ (*p16^Ink4aMSM^* allele knockout F_1_) and *p19^ArfFVB^*^/-^ (*p19^ArfMSM^* allele knockout F_1_) mice. They were treated with DMBA/TPA. As a result, a significantly higher number of papillomas developed in *p19^ArfFVB^*^/−^ mice. In contrast, the number of papillomas that developed in *p16^Ink4aFVB^*^/−^ mice did not markedly increase. Therefore, the *p19^ArfMSM^* allele appeared to confer greater resistance to the development of papilloma than the *p16^Ink4aMSM^* allele [60], which indicated that *p19^Arf^* was more likely to be the responsible gene for *Stmm3* than *p16^Ink4a^*. We then separately knocked out the MSM/M allele of *p19^Arf^* (*p19^ArfMSM^*) and FVB/N allele of *p19^Arf^* (*p19^ArfFVB^*) on (FVB/N × MSM/) F_1_ background and subjected these mice to DMBA/TPA skin carcinogenesis. The numbers of total papillomas and larger papillomas were significantly higher in MSM/Ms allele knockout mice (*p19^Arf FVB^*^/-^). These results indicated MSM/Ms allele of *p19^Arf^* more strongly inhibited papilloma genesis and growth than the FVB/N allele, confirming MSM/Ms allele was a resistance gene. We also showed that the p53 pathway was more efficiently activated by the *p19^ArfMSM^* allele than by the *p19^ArfFVB^* allele in vitro (Figure 4). Furthermore, novel polymorphisms in human *CDKN2A* that were near the SNP in mouse *Cdkn2a* were shown to be associated with the risk of human breast cancers. These results strongly suggested that linkage study started with MSM/Ms mice allowed us to identify the responsible gene for *Stmm3* locus, and finally proved its human orthologue functioned as a tumor resistance/susceptibility gene also in humans.

## 4. Closing Remarks

MSM/Ms mice were originally captured in Mishima, Japan in the 1970s. After long-term repeated sister-brother mating, the mice were established as a pure inbred mouse strain and have been utilized as a valuable genetic tool for mouse genetics for the past 40 years, mainly in Japan. MSM/Ms mice were originally used mainly for genetic mapping by SSLP markers because of their extensive polymorphism with standard inbred mouse strains. In the field of cancer biology, tumor cell lines were often established from F1 mice between MSM/Ms and inbred mice and used for LOH analysis. As SSLP markers were established, they were used for positional cloning of single trait mutants as well as multigenic traits, including cancer. In this review, we focused on radiation-induced lymphomas and chemically-induced skin tumors. A lot of other cancers and multigenic diseases were examined with MSM/Ms. However, a very limited number of studies reached particular responsible genes. In the majority of studies, genetic loci were mapped on chromosomes, but particular genes were not identified, because of the lack of genome information previously. In other words, a large number of interesting genes are still yet to be identified in MSM/Ms. Genetic resources are already established with MSM/Ms, such as the complete genome sequence information [21], a full set of mouse consomic strains [61], the BAC clone library [24], Microsatellite database [23], and ES cells [37]. Using currently available genetic tools developed from MSM/Ms will allow us to identify responsible genes and polymorphisms for complex traits including cancer, which were previously difficult to handle. 

## 5. Conclusions

Recently, new carcinogenesis models, such as organoids, were developed and next-generation sequencing of human samples was widely carried out. However, the benefit of mouse models is that all the experiments can be carried out in a situation where the immune system and the tumor microenvironment are maintained in the same way as in humans. The important thing is that several carcinogenesis models complement one another.

Finally, the integration of this information from carcinogenesis models and human GWAS information will facilitate the identification of human cancer susceptibility genes and polymorphisms, which will enable us to predict cancer risk and develop strategies for targeted therapy

## Figures and Tables

**Table 1:** Tumor types examined with MSM/Ms (MISHIMA/Mishima).

Tumor Induction	Tumor Types	References
Urethan	Lung tumors	[22]
PhIP	Intestinal tumors	[29]
Min mice	Intestinal tumors	[30]
MuLV	B-cell lymphomas	[31]
γ-ray	Thymic lymphomas	[32,33]
DEN	Hepatocellular carcinomas	[34]
MNU	Forestomach tumors	[35]
DMBA/TPA	Skin tumors	[36]

PhIP; 2-Amino-1-methyl-6-phenylimidazo (4,5-b) pyridine, Min; Multiple intestinal neoplasia, MuLV; Murine leukemia virus, DEN; Diethylnitrosamine, MNU; N-methyl-N-nitrosourea, DMBA; 7. 12-Dimethylbenz(a)anthracene, TPA; 12-O-Tetradecanoylphorbol-13-acetate.

**Table 2:** Tumor resistance is dependent on mouse strains.

Tumor Types	FVB/N	BALB/c	C57BL6/J	MSM/Ms
Resistance to radiation-induced lymphomas	++	++	+++	+++++
Resistance to chemically- induced skin tumors	+	++	+++	+++++

+ indicates the extent of tumor resistance. The more + means the more resistant.

## Data Availability

The data presented in this study are available on request from the corresponding author.
